# Progress Toward Global Eradication of Dracunculiasis, January 2020–June 2021

**DOI:** 10.15585/mmwr.mm7044a1

**Published:** 2021-11-05

**Authors:** Donald R. Hopkins, Adam J. Weiss, Sharon L. Roy, Sarah Yerian, Vitaliano A. Cama

**Affiliations:** ^1^The Carter Center, Atlanta, Georgia; ^2^World Health Organization Collaborating Center for Dracunculiasis Eradication, Division of Parasitic Diseases and Malaria, Center for Global Health, CDC.

Dracunculiasis (Guinea worm disease), caused by the parasite *Dracunculus medinensis*, is traditionally acquired by drinking water containing copepods (water fleas) infected with *D. medinensis* larvae, but in recent years also appears increasingly to be transmitted by eating fish or other aquatic animals. The worm typically emerges through the skin on a lower limb of the host 1 year after infection, causing pain and disability (*1*). There is no vaccine or medicine to prevent or medicine to treat dracunculiasis; eradication relies on case containment* to prevent water contamination and other interventions to prevent infection: health education, water filtration, treatment of unsafe water with temephos (an organophosphate larvicide), and provision of safe drinking water (*1*,*2*). The eradication campaign began in 1980 at CDC (*1*). In 1986, with an estimated 3.5 million cases^†^ occurring annually in 20 African and Asian countries^§^ (*3*), the World Health Assembly called for dracunculiasis elimination (*4*). The Guinea Worm Eradication Program (GWEP), led by The Carter Center and supported by the World Health Organization (WHO), UNICEF, CDC, and other partners, began assisting ministries of health in countries with endemic disease. With 27 cases in humans reported in 2020, five during January–June 2021, and only six countries currently affected by dracunculiasis (Angola, Chad, Ethiopia, Mali, South Sudan, and importations into Cameroon), achievement of eradication appears to be close. However, dracunculiasis eradication is challenged by civil unrest, insecurity, and epidemiologic and zoologic concerns. Guinea worm infections in dogs were first reported in Chad in 2012. Animal infections have now overtaken human cases, with 1,601 reported animal infections in 2020 and 443 during January–June 2021. Currently, all national GWEPs remain fully operational, with precautions taken to ensure safety of program staff and community members in response to the COVID-19 pandemic. Because of COVID-19, The Carter Center convened the 2020 and 2021 annual GWEP Program Managers meetings virtually, and WHO’s International Commission for the Certification of Dracunculiasis Eradication met virtually in October 2020. Since 1986, WHO has certified 199 countries, areas, and territories dracunculiasis-free. Six countries are still affected: five with endemic disease and importations into Cameroon. Seven countries (five with endemic dracunculiasis, Democratic Republic of the Congo, and Sudan) still lack certification (*4*). The existence of infected dogs, especially in Chad, and impeded access because of civil unrest and insecurity in Mali and South Sudan are now the greatest challenges to interrupting transmission. This report describes progress during January 2020–June 2021 and updates previous reports (*2*,*4*,*5*).

In 2020, Angola, Cameroon, Chad, Ethiopia, Mali, and South Sudan reported 27 cases in humans; Cameroon, Chad, Ethiopia, and Mali reported 1,601 infected animals (mostly domestic dogs), compared with 54 cases in humans and 2,000 infections in animals in 2019 (Table 1). During January–June 2021, five cases were reported in Chad (four) and Ethiopia (one), and 443 infected animals were reported in Chad (441) and Mali (two), compared with 19 cases and 1,115 animal infections during January–June 2020. This difference equates to a 74% reduction in human cases and a 60% reduction in animal infections during this 6-month period in 2021 compared with 2020. During January–June 2021, CDC received 13 specimens from humans for morphologic or molecular identification, including five that were laboratory-confirmed *D. medinensis*^¶^ (Table 2), compared with 44 received and 21 (41%) confirmed during January–June 2020. During the first 6 months of 2021, CDC received 36 animal specimens, six (17%) of which were confirmed, compared with 19 received and five confirmed during January–June 2020. *D. medinensis* worms from animals are genetically indistinguishable from those removed from humans (*6*). This activity was reviewed by CDC and was conducted consistent with applicable federal law and CDC policy.**

**TABLE 1:** Number of reported indigenous dracunculiasis cases in humans and infections in animals, by country — worldwide, January 2019–June 2021

Country	No. (% Contained)		% Change Jan–Dec 2019 to Jan–Dec 2020	No. (% Contained)		% Change Jan–Jun 2020 to Jan–Jun 2021
	Jan–Dec 2019	Jan–Dec 2020		Jan–Jun 2020	Jan–Jun 2021	
**Human cases**						
Angola	1 (0)	1 (0)	0	1 (0)	0 (—)	–100
Cameroon	0 (—)	1 (0)*	—	1 (0)	0 (—)	–100
Chad	49 (53)	12 (42)	–75	9 (44)	4 (75)	–56
Ethiopia	0 (—)	11 (100)	—	7 (100)	1 (100)	–86
Mali^†^	0 (—)	1 (0)	—	1 (0)	0 (—)	–100
South Sudan	4 (50)	1 (100)	–75	0 (—)	0(—)	—
**Total**	**54 (52)**	**27 (63)**	**–51**	**19 (58)**	**5 (80)**	**–74**
**Animal infections** ^§^						
Angola	1 (0)	0 (—)	–100	0 (—)	0 (—)	—
Cameroon	0 (—)	6 (100)*	—	6 (100)	0 (—)	–100
Chad	1,982 (76)	1,571 (81)	–21	1,106 (79)	441 (60)	–61
Ethiopia	8 (25)	15 (73)	88	3 (33)	0 (—)	–100
Mali^†^	9 (67)	9 (56)	0	0 (—)	2 (100)	—
South Sudan	0 (—)	0 (—)	—	0 (—)	0 (—)	—
**Total**	**2,000 (76)**	**1,601 (81)**	**–20**	**1,115 (79)**	**443 (60)**	**–60**

* Cameroon reported one human case, five infected dogs, and one infected cat in 2020 in villages <3 miles (<5 km) from the Chad-Cameroon border that were likely infected in Chad because the affected villages included families living on both sides of the border.

^†^ Civil unrest and insecurity since a coup d'état in April 2012 continued to constrain Guinea Worm Eradication Program operations (e.g., supervision, surveillance, and interventions) in Gao, Kidal, Mopti, Segou, and Timbuktu regions.

^§^ In Chad, primarily dogs, some cats; in Ethiopia, dogs, cats, and baboons; in Mali, dogs and one cat (2019); in Angola, one dog; in Cameroon, dogs and one cat.

**TABLE 2:** Characteristics of specimens from humans and animals received at CDC for laboratory diagnosis of *Dracunculus medinensis* — January 2020–June 2021

Specimens received at CDC	Jan–Dec 2020	Jan–Jun 2021
**From humans**		
**No. received**	91	13
**No. laboratory confirmed as *D. medinensis* (%)**	39 (43)	5 (38)
**Country of origin**		
Angola	1 (3)	—*
Cameroon	1 (3)	—*
Chad	12 (31)	4 (80)
Ethiopia	23 (59)	1 (20)
Mali	1(3)	—*
South Sudan	1 (3)	—*
**No. ruled out as *D. medinensis* (%)**	52 (57)	8 (62)
**Other laboratory diagnoses**		
Free-living nematode^†^	19 (37)	2 (25)
Onchocerca	3 (6)	—^§^
Other parasitic nematode^†^	9 (17)^¶^	—^§^
Sparganum	10 (19)	3 (38)
Animal-origin tissue	5 (10)	2 (25)
Plant material	—^§^	—^§^
Other worms	3 (6)**	—^§^
Other	1 (2)^††^	—^§^
Other *Dracunculus* sp.	2 (4)	—^§^
Unknown origin	—^§^	1 (13)
**From animals**		
**No. received**	92	36
**No. laboratory confirmed as *D. medinensis* (%)**	43 (47)	6 (17)
**Country/Species of origin**		
Cameroon	7^§§^	—^¶¶^
Dog	6 (86)^§§^ֻ	—^¶¶^
Cat	1 (14)	—^¶¶^
Central African Republic	—^¶¶^	1
Dog	—^¶¶^	1 (100)
Chad	7	3
Cat	4 (57)	—^¶¶^
Dog	3 (43)	2 (67)
Wildcat	—^¶¶^	1 (33)
Ethiopia	19	—^¶¶^
Baboon	5 (26)	—^¶¶^
Cat	9 (47)	—^¶¶^
Dog	5 (26)	—^¶¶^
Mali	10	2
Cat	—^¶¶^	—^¶¶^
Dog	10 (100)	2 (100)
**No. ruled out as *D. medinensis* (%)**	49 (53)	30 (83)
**Other laboratory diagnoses**		
Free-living nematode^†^	14 (29)	2 (7)
Other parasitic nematode^†^	24 (49)***	22 (73)***
Animal-origin tissue	2 (4)	1 (3)
Other worms	5 (10)^†††^	2 (7)
Other	3 (6)^§§§^	—^§^
Plant material	1 (2)	3 (10)

* Specimens did not come from this country.

† The category “Free-living nematodes” primarily included adult Mermithidae and other nematodes identified as belonging to nonparasitic taxa. “Other parasitic nematodes” included non-Onchocerca nematodes identified as belonging to parasitic taxa.

^§^ No specimens received this diagnosis.

^¶^ “Other parasitic nematodes” submitted in association with cases in humans in 2020 included *Eustrongyloides* sp. (one) and filarial nematodes not fully identified (four).

** “Other worms” submitted in association with one case in a human in 2020 included an annelid (one); a horsehair (Gordian) worm (one); a specimen vial that contained two Acanthocephala not identified further and one Toxocara; and one nematode not able to be identified further.

^††^ The “Other” specimen submitted in association with one case in a human in 2020 included a small (approximately 15 cm) blind snake (infraorder Scolecophidia).

^§§^ Two worms were submitted from one dog.

^¶¶^ No specimen came from that country or that species.

*** “Other parasitic nematodes” submitted in association with animal cases in 2020 included *Dirofilaria* sp. (two), *Filaria* sp. (11), *Physaloptera* sp. (two), *Protospirura* sp. (one), *Skrjabinodera* sp. (three), *Spirocerca* sp. (one), spirurid nematodes (two), and *Toxocara cati* (two). Submissions during January–June 2021 included *Eustrongyloides* sp. (one), *Dirofilaria* sp. (two), *Skrjabinodera* sp. (one), filarial nematodes not identified to genus (16), a worm belonging to the Gnathostoma taxon (one), and a spirurid nematode not identified to genus (one).

^†††^ “Other worms” submitted in association with one case in an animal in 2020 included an Acanthocephala not identified further (one), cestodes not identified further (two), nematode not identified further (one), and a horsehair worm (one).

^§§§^ The “Other” specimens submitted in association with animal cases in 2020 were small snakes (two) and a fly larva (one).

In affected countries, the national GWEP receives monthly case reports from supervised volunteers in villages under active surveillance^††^ (Table 3). Villages where endemic transmission has ended (i.e., zero cases or animal infections reported for ≥12 consecutive months) are kept under active surveillance for 2 additional years. WHO certifies a country as dracunculiasis-free after adequate nationwide surveillance for ≥3 consecutive years with no indigenous cases or animal infections.^§§^

**TABLE 3:** Reported dracunculiasis cases in humans and infections in animals, surveillance, and status of local interventions in villages with endemic disease, by country — worldwide, 2020

Human cases/Surveillance/Intervention status	Country					
	Angola	Chad*	Ethiopia	Mali^†^	South Sudan	Total
**Reported human cases**						
No. indigenous, 2020	1	13^§^	11	1	1	27
No. imported,^¶^ 2020	0	0	0	0	0	0
% Contained,** 2020	0	42	100	0	100	63
% Change in indigenous human cases in villages/localities under surveillance, same period 2019 and 2020	0	–76	—	—	–75	–50
**Reported animal cases**						
No. indigenous, 2020	0	1,577^††^	15	9	0	1,601
No. imported,^§§^ 2020	0	0	0	0	0	0
% Contained,** 2020	0	81	73	56	0	81
% Change in indigenous animal infections in villages/localities under surveillance, same period 2019 to 2020	–100	–19	88	50	—	–18
**Villages under active surveillance, 2020**						
No. of villages	54	2,332	190	2,699	851	6,847
% Reporting monthly	—***	99	100	100	98	99
No. reporting ≥1 human case	1	9	5	1	1	17
No. reporting only imported^§§^ human cases	0	0	1	1	1	3
No. reporting indigenous human cases	1	9	4	0	0	14
No. reporting ≥1 animal infection	0	428	12	5	0	445
No. reporting only imported^§§^ animal infections	0	0	1	1	0	2
No. reporting indigenous animal infections	0	428	11	4	0	443
**Status of interventions in villages with endemic human dracunculiasis, 2020**						
No. villages with endemic human dracunculiasis, 2019–2020	2	2	4	0	2	10
% Reporting monthly^¶¶^	—***	100	100	—^†††^	100	100
% Filters in all households^¶¶^	100	0	100	—^†††^	100	100
% Using temephos^¶¶^	0	100	100	—^†††^	100	80
% With ≥1 source of safe water^¶¶^	50	0	75	—^†††^	50	—
% Provided health education^¶¶^	100	100	100	—	100	100
**Status of interventions in villages with endemic animal dracunculiasis, 2020**						
No. villages with endemic animal dracunculiasis, 2019–2020	1	267	14	10	0	292
% Reporting monthly^¶¶^	—***	99	100	100	—	99
% Using temephos^¶¶^	0	84	100	100	—	85
% Provided health education^¶¶^	100	99	100	100	—	99

* Participants at the annual Chad Guinea Worm Eradication Program review meeting in November 2014 adopted “1+ case village” as a new description for villages in Chad affected by cases in humans of Guinea worm disease or dogs infected with Guinea worms and defined it as “a village with one or more indigenous and/or imported cases of Guinea worm infections in humans, dogs, and/or cats in the current calendar year and/or previous year.”

^†^ Civil unrest and insecurity since a coup d'état in April 2012 continued to constrain Guinea Worm Eradication Program operations (e.g., supervision, surveillance, and interventions) in Gao, Kidal, Mopti, Segou, and Timbuktu regions.

^§^ Twelve cases were reported from Chad in 2020. One case was reported from Cameroon in 2020 in a village along the Chad-Cameroon border which is believed to have been acquired in Chad because the affected villages included families living on both sides of the Cameroon-Chad border.

^¶^ Imported from another country.

** Transmission from a patient with dracunculiasis is contained only if all of the following conditions are met for each emerged worm: 1) the infected patient is identified ≤24 hours after worm emergence; 2) the patient has not entered any water source since the worm emerged; 3) a village volunteer or other health care provider has managed the patient properly by cleaning and bandaging the lesion until the worm has been fully removed manually and by providing health education to discourage the patient from contaminating any water source (if two or more emerging worms are present, transmission is not contained until the last worm is removed); 4) the containment process, including verification of dracunculiasis, is validated by a Guinea Worm Eradication Program supervisor within 7 days of emergence of the worm; and 5) temephos is used to treat potentially contaminated surface water if any uncertainty about contamination of these sources of drinking water exists or if such a source of drinking water is known to have been contaminated.

^††^ Chad reported 1,571 animal cases in 2020. Six cases in animals were reported from Cameroon in 2020, all in villages along the Chad-Cameroon border; these are believed to have been acquired in Chad.

^§§^ Imported from another in-country disease-endemic village.

^¶¶^ The denominator is number of villages with endemic human or animal dracunculiasis reported during 2019–2020.

*** By the end of 2020, Angola established active surveillance in 54 villages, but a system for regular data reports from each village was not yet established.

^†††^ The case in a human in Mali was imported from another location within Mali. The village of detection is not considered endemic because the case was not indigenous to the village of detection. Therefore, interventions such as monthly reporting, filter distribution, temephos use, and safe water source counting were not implemented in the village of detection and were not applicable.

## Country Reports

**Angola.** Details of the unexpected discovery of dracunculiasis in three humans during 2018–2020 with no history of foreign travel and one dog in 2019 have been described previously (*4*). Despite ongoing active surveillance in 54 communities and routine integrated case searches (e.g., during National Immunization Days), no case or infected dog was detected from January 2021 through June 2021. Angola offers a US$450 equivalent reward for reporting an infected human or animal. Provisional DNA analysis of Angola’s Guinea worm specimens yielded no clear link to another *D. medinensis* population.

**Chad**. Chad reported 12 cases in 10 villages in 2020. During the first half of 2021, Chad reported four cases in humans, 56% fewer cases than the nine reported during January–June 2020.

During 2020, Chad reported 1,508 dog and 63 cat infections, compared with 1,935 dog and 47 cat infections in 2019 (Table 1). During January–June 2021, 60% fewer infected dogs (428) and 48% fewer infected cats (13) were reported than during the same period of 2020 (1,081 dogs and 25 cats). Transmission of *D. medinensis* to humans and animals is hypothesized to occur from eating inadequately cooked fish, other aquatic transport, or paratenic hosts (hosts in which the larval parasite does not develop) (*7*). The Carter Center is helping Chad’s Ministry of Health implement active village-based surveillance for animal and human infections in 2,336 at-risk villages as of June 2021, an increase of four villages since December 2020 (Table 3). Since June 2017, approximately 81% of households sampled monthly in communities at risk were burying fish entrails; 83% and 81% of infected dogs were tethered (contained) in 2020 and during January–June 2021, respectively. Water treatment with temephos reached 75% of 408 villages with dog or human infections by December 2020 and 87% of 182 villages by June 2021. In December 2020, 49% of villages reporting infected dogs or humans had at least one source of copepod-free drinking water.

In areas under surveillance in Chad, 83% of residents surveyed in 2020 knew of the cash rewards for reporting human or animal infections; 92% of those surveyed in January–June 2021 knew of the rewards. Surveillance generated 61,341 reports about possible human or dog infections during January–June 2021 compared with 50,893 during the same period in 2020.

**Cameroon**. Cameroon reported one case, five infected dogs, and one infected cat in 2020 in villages less than 3 miles (5 km) from the Chad-Cameroon border that were likely infected in Chad, because the affected villages included families living on both sides of the border; Cameroon reported no infected humans or animals through June 2021.

**Ethiopia**. Ethiopia reported 11 cases in 2020, and one case during January–June 2021. The 2020 cases were in villagers exposed to a shared source of contaminated drinking water near Duli village in Gambella Region. During 2020, Ethiopia reported three infected dogs, eight cats, and four baboons, all in Gog district of Gambella Region, compared with two infected dogs and six baboons in 2019. During January–June 2021, Ethiopia reported one infected dog in Gog, compared with one infected dog and two baboons, also in Gog, during January–June 2020. Since 2017, The Carter Center has supported Ethiopia’s public health and wildlife authorities in a baboon and dog epidemiology project (*2*).

Since 2021, the Ethiopia Dracunculiasis Eradication Program has had 198 villages under active surveillance. The program applies temephos monthly to all water sources known to have been used by humans in the at-risk area of Gog, and, since April 2018, it has supported villager-initiated constant tethering of approximately 1,914 dogs and cats in villages where most infected animals were detected in recent years to prevent their exposure to water sources in adjacent forests where transmission apparently occurs (*2*). In 2018, Ethiopia increased rewards for reporting human dracunculiasis cases to US$360 equivalent and for reporting and tethering infected animals to US$40. In 2020, 92% of persons surveyed in active surveillance areas knew of the rewards; in January–May 2021, 96% were aware.

**Mali.** Mali reported one case in a human in 2020 after 4 consecutive years of zero cases; no cases in humans were reported during January–June 2021 compared with one during the same period in 2020. During 2020, nine infected dogs were reported, compared with eight dogs and one cat in 2019. During the first half of 2021, Mali reported two infected dogs, compared with no infected animals during January–June 2020. Six infected dogs identified in 2020 were detected in Segou Region; three were detected in adjacent Djenne district of Mopti Region. Segou Region is accessible to the program, but the dogs sold in Segou were bred and apparently became infected in areas of Mopti Region that have remained inaccessible since 2012. The two infected dogs identified during January–June 2021 were detected in Segou Region. In 2020, Mali had 2,699 villages under active surveillance. The reward for reporting a case in a human was US$340 equivalent and US$20 equivalent for reporting and tethering an infected animal. In areas under active surveillance in 2020, 89% of persons queried knew of the rewards for reporting an infected person or animal; 95% of persons queried in January–May 2021 were aware.

**South Sudan.** South Sudan reported one case in a human in the latter half of 2020, compared with four in 2019, and none during January–June 2021. Only one infected animal has been reported (a dog in the same household as an infected person in 2015). Extreme population mobility of cattle herders and others is a special challenge in addition to sporadic insecurity. By December 2020, South Sudan’s GWEP had 851 villages under active surveillance. The reward for reporting a case of dracunculiasis is approximately US$280 equivalent (US$26 for animals). A 2020 survey of residents found 93% of respondents knew of the reward for reporting an infected person.

## Discussion

After a decade with no reported cases, Chad reported 10 indigenous cases in humans in 2010; Guinea worm infections in domestic dogs were reported for the first time in 2012, mostly from communities along the Chari River in a pattern affecting many dogs and few humans that remains peculiar to Chad (*7*). During January 2020–June 2021, Chad reported 98% of the world’s remaining *D. medinensis* infections, 94% of which were in dogs. Stopping transmission in Chadian dogs is now the biggest challenge to the eradication program. The challenge is being addressed through innovative interventions and research supported by The Carter Center and CDC involving multiple research institutions to help understand the unusual epidemiology of dracunculiasis in the remaining countries and assessing antihelminthic treatment of dogs (*8*). Researchers from the University of Georgia have shown in the laboratory that fish can serve as transport hosts for *Dracunculus* spp. and that *D. medinensis* can use frogs as paratenic hosts; *Dracunculus* larvae have been recovered from multiple wild frogs in Chad (*9*,*10*). If the hypothesis that the parasite’s life cycle in Chad involves a transport or paratenic host is correct (*10*), increased active surveillance, proactive containment of dogs, temephos application, and fish entrail burial should reduce transmission. The 56% reduction in cases in humans and 61% reduction in animals in Chad in January–June 2021 suggests that is now happening.

Chad has offered a US$100 equivalent reward for reporting a confirmed human dracunculiasis case since 2010 and a reward of US$20 equivalent for reporting and tethering a confirmed infected dog since 2015. All reports must be corroborated by supervisors. Since October 2013, Chad’s GWEP has urged villagers to cook their fish well, bury fish entrails, and prevent animals from eating them. In 2014, volunteers began persuading villagers to tether dogs with signs of dracunculiasis (e.g., blisters or subcutaneous worms) until the worms emerged to prevent water contamination. In 2017, the program began applying temephos monthly to small ponds in villages with the most infected dogs and launched a nationwide communication campaign to increase awareness about the rewards and how to prevent Guinea worm infections. In March 2020, Chad launched a new strategy to tether all dogs proactively during the 4 months of peak dracunculiasis incidence in the 120 villages that reported five or more infections in 2019, an effort now being scaled up to all villages reporting one or more infections.

In 2020, Mali reported its first case in a human in over 4 years, and Ethiopia reported its first cases in humans in over 2 years. Continued endemic transmission among a few dogs and cats in Mali as well as baboons in Ethiopia appears to be geographically limited. Insecurity is still the main obstacle to stopping transmission in Mali. The ecologic study of baboons and proactive tethering of dogs in Gog district might clarify the dynamics of residual infections in Ethiopia.

If security remains adequate, South Sudan is poised to achieve zero-case status soon because of strong technical leadership, strong governmental support, and no animal infections. Finding three confirmed cases in humans and one infected dog in Angola in 2018–2020 and none so far in 2021 suggests that the problem there is limited.

Identification of dracunculiasis cases in a Cameroonian border village during 2018–2020 highlights the risks for exportation from Chad and the need for ongoing active surveillance in neighboring Cameroon and the Central African Republic. Common source waterborne outbreaks in Ethiopia in 2020 highlight the need for safe drinking water wherever dracunculiasis occurs. The current prominence of infections in domestic dogs and cats requires increased measures to limit those animals’ access to waste from discarded fish and other aquatic animals.

**Summary**

**What is already known about this topic?**

Cases of dracunculiasis (Guinea worm disease) have decreased from an estimated 3.5 million in 1986 to 27 in 2020. Emergence of Guinea worm infections in dogs in 2012 has complicated eradication efforts.

**What is added by this report?**

With 27 cases in humans reported in 2020, five during January–June 2021, and only six countries currently affected by dracunculiasis (Angola, Chad, Ethiopia, Mali, South Sudan, and importations into Cameroon), achievement of eradication appears to be close.

**What are the implications for public health practice?**

Existence of infected dogs, especially in Chad, and impeded access because of civil unrest and insecurity in Mali and South Sudan are now the greatest challenges to interrupting transmission.

## Footnotes

* Transmission from a patient with dracunculiasis is considered to be contained only if all of the following conditions are met for each emerging worm: 1) the infected patient is identified ≤24 hours after worm emergence; 2) the patient has not entered any water source since the worm emerged; 3) a village volunteer or other health care provider has managed the patient properly; 4) the containment process, including verification of dracunculiasis, is validated by a Guinea Worm Eradication Program supervisor within 7 days of emergence of the worm; and 5) the approved chemical temephos (Abate) is used to treat known or potentially contaminated surface water. Proper patient management includes cleaning and bandaging the lesion until the worm has been fully removed manually and by providing health education to discourage the patient from contaminating any water source. If two or more emerging worms are present, transmission is not contained until the last worm is removed. Similar criteria are in place for the containment of animal infections.

† A dracunculiasis case is defined as an infection occurring in a person exhibiting a skin lesion or lesions with emergence of one or more worms that is laboratory-confirmed as *Dracunculus medinensis* at CDC. Because *D. medinensis* has a 10–14-month incubation period, each infected person is counted as having a case only once during a calendar year.

§ Initially 20 countries, but the former country of Sudan officially separated into two countries (Sudan and South Sudan) on July 9, 2011.

¶ Specimens are laboratory-confirmed as *D. medinensis* at CDC by morphologic examination under a microscope or polymerase chain reaction assay. https://www.cdc.gov/dpdx/dxassistance.html

** 45 C.F.R. part 46.102(l)(2), 21 C.F.R. part 56; 42 U.S.C. Sect. 241(d); 5 U.S.C. Sect. 552a; 44 U.S.C. Sect. 3501 et seq.

†† Villages under active surveillance are those that have endemic dracunculiasis or are at high risk for importation. Active surveillance involves daily searches of households by village volunteers (supported by their supervisors) for persons or animals with signs of dracunculiasis. An imported case in a human or animal infection is one resulting from ingestion of contaminated water or transport/paratenic host in a place other than the community where the case or infection is detected and reported. Since 2012, no internationally imported cases or infections have been reported except in Cameroon.

§§ An indigenous dracunculiasis case in a human or animal infection is defined as an infection consisting of a skin lesion or lesions with emergence of one or more Guinea worms in a person or animal who had no history of travel outside their residential locality during the preceding year.
